# Geographical Disparities in Research Misconduct: Analyzing Retraction Patterns by Country

**DOI:** 10.2196/65775

**Published:** 2025-01-14

**Authors:** Paul Sebo, Melissa Sebo

**Affiliations:** 1 University Institute for Primary Care University of Geneva Geneva Switzerland; 2 Faculty of Medicine University of Geneva Geneva Switzerland

**Keywords:** affiliation, country, fraud, integrity, misconduct, plagiarism, publication, research, retraction, ethical standards, ethics, research misconduct, literature

## Abstract

This study examines disparities in research retractions due to misconduct, identifying countries with the highest retraction counts and those disproportionately represented relative to population and publication output. The findings emphasize the need for improved research integrity measures.

## Introduction

Retractions are essential for maintaining scientific integrity, especially in cases of research misconduct [1-4]. Data from 2013 to 2015 show that retraction rates vary by country due to differences in research culture, regulations, and publication pressures [3]. Understanding these variations is vital to identifying systemic issues in research integrity.

We examined the countries with the highest numbers of retractions due to misconduct, analyzing both absolute counts and proportions relative to population size and publication output. Our goal is to show the geographical distribution of research misconduct and identify countries disproportionately represented in retraction statistics.

## Methods

### Data Source and Analysis

We used data from the SCImago Journal & Country Rank (SJR), based on Scopus data, to identify the top 100 countries by publication volume from 1996 to 2023—both overall and within the field of medicine. This approach was previously used to identify the most productive countries [5]. Retraction counts (1996-2023) were obtained from the Retraction Watch (RW) database, isolating retractions due to misconduct. Misconduct was defined according to criteria previously established [6] and detailed in Multimedia Appendix 1.

Both SJR and RW consider all authors listed on an article, regardless of position, when attributing publications and retractions, respectively, to a country. They use full counts, equally attributing publications and retractions to all listed countries. For each country, we analyzed absolute retraction counts and proportions weighted by population size (United Nations 2023 data) [7] and by publication output, calculated by dividing retraction count by total publication count. Data were collected independently by two researchers (PS and MS), with any discrepancies resolved through discussion.

### Ethical Considerations

As this study did not involve the collection of personal health-related data, it did not require ethical review in accordance with Swiss legislation.

## Results

**Figure 1:**
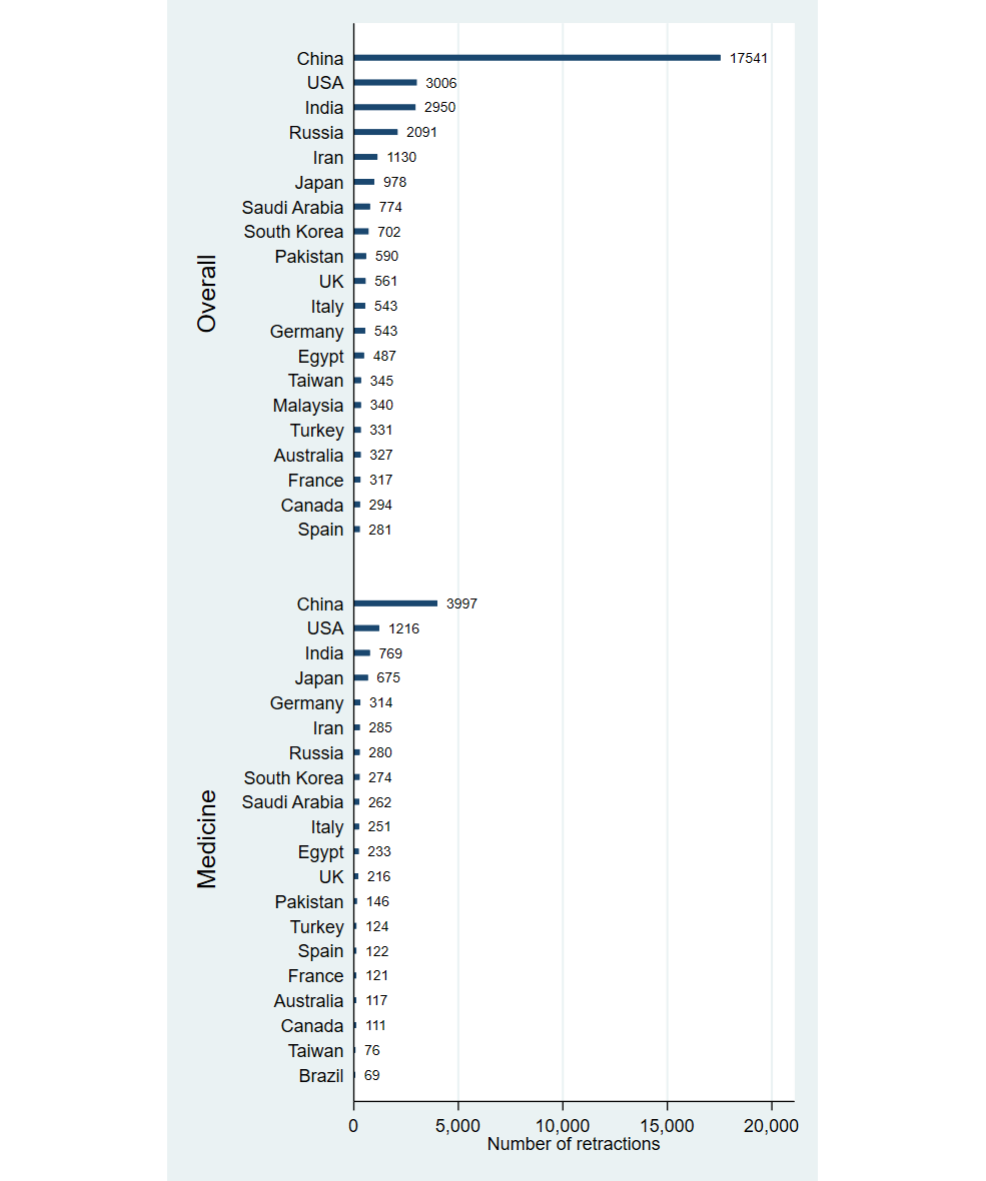
Number of retractions by country (1996-2023) based on overall publications and those in the field of medicine.

**Figure 2:**
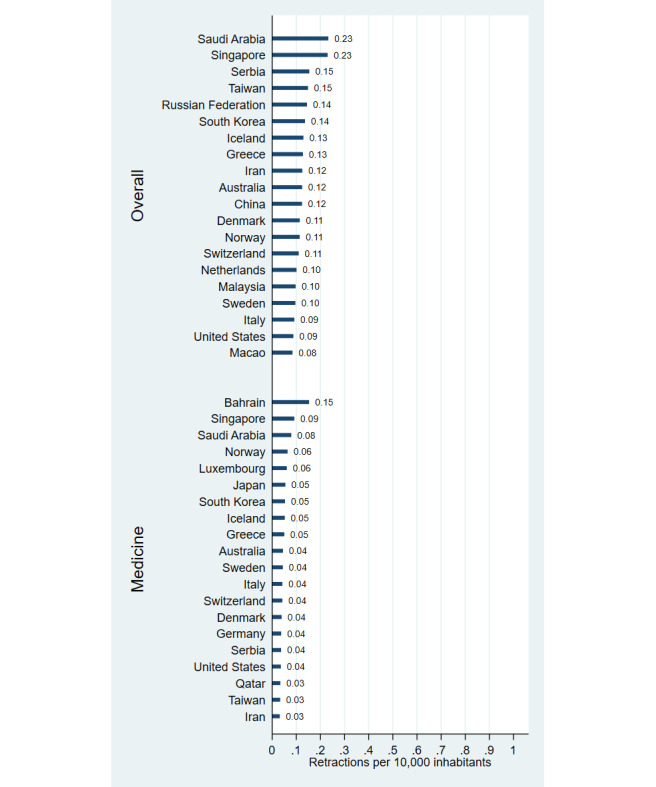
Number of retractions per 10,000 inhabitants by country (1996–2023), based on overall publications and those in the field of medicine.

**Figure 3:**
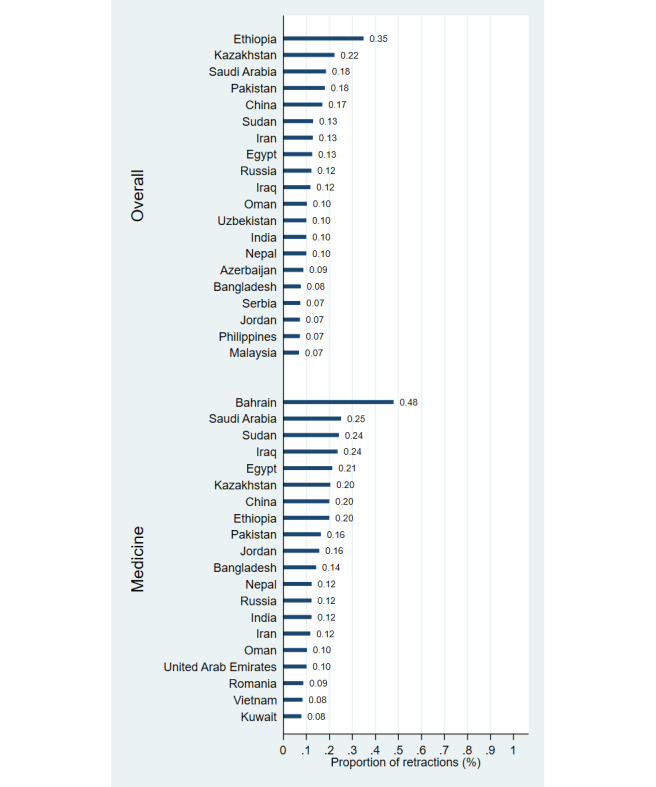
Proportion of retractions relative to publication output, by country (1996-2023), based on overall publications and those in the field of medicine.

Detailed results are available in Multimedia Appendix 1 and Figures 1-3. The US, China, the UK, Germany, and Japan are the top 5 productive countries in terms of both overall publications and publications in medicine. Across the 100 countries analyzed, there were 37,858 retractions out of 79,645,579 publications overall (0.048%), and 10,890 retractions out of 23,175,369 publications in medicine (0.047%).

Our data suggests that China leads in retractions, far surpassing the US with more than five times as many retractions overall (17,541 vs 3006) and three times as many in medicine (3997 vs 1216). India ranks third with 2950 retractions overall and 769 in medicine. When adjusting for population size, Saudi Arabia, Singapore, Serbia, Taiwan, and Russia have the highest retraction rates overall, while Bahrain, Singapore, Saudi Arabia, Norway, and Luxembourg lead in medicine. Asian, Middle Eastern, and European countries are notably overrepresented in population-adjusted retractions. The highest retraction proportions relative to overall publication output were found in Ethiopia (0.35%), Kazakhstan (0.22%), Saudi Arabia (0.19%), Pakistan (0.18%), and China (0.17%), and in Bahrain (0.48%), Saudi Arabia (0.25%), Sudan (0.24%), Iraq (0.24%), and Egypt (0.21%) when evaluating the medicine subset, with overrepresentation among Asian, Middle Eastern, and African countries.

## Discussion

Our findings reveal that China, the US, and India have the highest numbers of retractions due to misconduct, with China particularly overrepresented. Retractions are disproportionately high in several Asian, Middle Eastern, and European countries when adjusted for population, as well as in several Asian, Middle Eastern, and African countries when adjusted for publication output. These results highlight regional disparities in research integrity.

These findings align with previous research identifying China, the US, and India as leaders in retraction numbers [2,3], with China’s prominence especially noticeable in recent years [8-10]. The overrepresentation of retractions among countries with emerging research sectors, as observed in this study, reflects challenges such as weaker oversight and high publication pressure [1-3]. Measures like stricter peer review, automated plagiarism detection, open data sharing, and pre-registration of studies can help mitigate misconduct and improve oversight.

Limitations of this study include reliance on the RW database, which may miss some misconduct cases, and a primary focus on medicine, potentially overlooking trends in other disciplines. Nonetheless, RW remains a trusted source for aggregated retraction data [8], with findings consistent across both overall and medicine-specific data. Additionally, differences between SJR (Scopus-based) publication data and RW’s broader retraction sources may slightly impact proportion calculations but are unlikely to affect key findings

In conclusion, this study underscores substantial geographical disparities in research misconduct, emphasizing the need for improved oversight and ethical standards, especially in regions with growing research sectors. Our findings contribute to ongoing discussions on the reliability of scientific research and the importance of global efforts to address misconduct.

## Appendix

Multimedia Appendix 1 Numbers and proportions of retractions by country; retractions per 10,000 inhabitants by country (1996-2023); and criteria for retraction used in the Retraction Watch database.

## Abbreviations

RW: Retraction Watch
SJR: SCImago Journal & Country Rank
