# Supplementary material for: Geographical Disparities in Research Misconduct: Analyzing Retraction Patterns by Country
Source: J Med Internet Res. 2025 Jan 14;27:e65775. doi: 10.2196/65775 (PMC11775486; doi:10.2196/65775)
Supplement: Multimedia Appendix 1 [file jmir_v27i1e65775_app1.docx]

**Table 1. Number and proportion of retractions by country, and retractions per 10,000 inhabitants by country (1996–2023). Countries sorted by total publication count.**

| Rank | Country | Region | Number of publications | Number of retractions | Retraction rate (%) | Population | Retractions per 10,000 inhabitants |
| --- | --- | --- | --- | --- | --- | --- | --- |
| 1 | United States | Northern America | 16047770 | 3006 | 0,0187 | 343477000 | 0,0875 |
| 2 | China | Asiatic Region | 10372322 | 17541 | 0,1691 | 1422580000 | 0,1233 |
| 3 | United Kingdom | Western Europe | 4778980 | 561 | 0,0117 | 68683000 | 0,0817 |
| 4 | Germany | Western Europe | 4104599 | 543 | 0,0132 | 84548200 | 0,0642 |
| 5 | Japan | Asiatic Region | 3482279 | 978 | 0,0281 | 124371000 | 0,0786 |
| 6 | India | Asiatic Region | 2970196 | 2950 | 0,0993 | 1438070000 | 0,0205 |
| 7 | France | Western Europe | 2784321 | 317 | 0,0114 | 66438800 | 0,0477 |
| 8 | Italy | Western Europe | 2525870 | 543 | 0,0215 | 59499500 | 0,0913 |
| 9 | Canada | Northern America | 2426840 | 294 | 0,0121 | 39299100 | 0,0748 |
| 10 | Australia | Pacific Region | 2009795 | 327 | 0,0163 | 26451100 | 0,1236 |
| 11 | Spain | Western Europe | 1986724 | 281 | 0,0141 | 47911600 | 0,0586 |
| 12 | Russian Federation | Eastern Europe | 1722547 | 2091 | 0,1214 | 145440000 | 0,1438 |
| 13 | South Korea | Asiatic Region | 1604519 | 702 | 0,0438 | 51748700 | 0,1357 |
| 14 | Brazil | Latin America | 1427852 | 154 | 0,0108 | 211141000 | 0,0073 |
| 15 | Netherlands | Western Europe | 1343844 | 182 | 0,0135 | 18092500 | 0,1006 |
| 16 | Switzerland | Western Europe | 1001958 | 97 | 0,0097 | 8870560 | 0,1094 |
| 17 | Poland | Eastern Europe | 964968 | 115 | 0,0119 | 38762800 | 0,0297 |
| 18 | Turkey | Middle East | 919863 | 331 | 0,0360 | 87270500 | 0,0379 |
| 19 | Sweden | Western Europe | 908607 | 101 | 0,0111 | 10551500 | 0,0957 |
| 20 | Iran | Middle East | 886359 | 1130 | 0,1275 | 90608700 | 0,1247 |
| 21 | Taiwan | Asiatic Region | 880380 | 345 | 0,0392 | 23317100 | 0,1480 |
| 22 | Belgium | Western Europe | 739211 | 75 | 0,0101 | 11712900 | 0,0640 |
| 23 | Denmark | Western Europe | 571552 | 68 | 0,0119 | 5948140 | 0,1143 |
| 24 | Austria | Western Europe | 557479 | 38 | 0,0068 | 9130430 | 0,0416 |
| 25 | Israel | Middle East | 514345 | 54 | 0,0105 | 9256310 | 0,0583 |
| 26 | Malaysia | Asiatic Region | 502815 | 340 | 0,0676 | 35126300 | 0,0968 |
| 27 | Portugal | Western Europe | 485426 | 68 | 0,0140 | 10430700 | 0,0652 |
| 28 | Mexico | Latin America | 483821 | 52 | 0,0107 | 129740000 | 0,0040 |
| 29 | Czech Republic | Eastern Europe | 468680 | 67 | 0,0143 | 10809700 | 0,0620 |
| 30 | Finland | Western Europe | 466403 | 30 | 0,0064 | 5601180 | 0,0536 |
| 31 | Norway | Western Europe | 464965 | 63 | 0,0135 | 5519170 | 0,1141 |
| 32 | Hong Kong | Asiatic Region | 461423 | 52 | 0,0113 | 7442730 | 0,0699 |
| 33 | South Africa | Africa | 448391 | 65 | 0,0145 | 63212400 | 0,0103 |
| 34 | Greece | Western Europe | 447972 | 130 | 0,0290 | 10242900 | 0,1269 |
| 35 | Singapore | Asiatic Region | 432121 | 133 | 0,0308 | 5789090 | 0,2297 |
| 36 | Saudi Arabia | Middle East | 419090 | 774 | 0,1847 | 33264300 | 0,2327 |
| 37 | Egypt | Africa/Middle East | 389675 | 487 | 0,1250 | 114536000 | 0,0425 |
| 38 | Indonesia | Asiatic Region | 376908 | 88 | 0,0233 | 281190000 | 0,0031 |
| 39 | New Zealand | Pacific Region | 342097 | 23 | 0,0067 | 5172840 | 0,0445 |
| 40 | Pakistan | Asiatic Region | 328610 | 590 | 0,1795 | 247504000 | 0,0238 |
| 41 | Ireland | Western Europe | 309576 | 38 | 0,0123 | 5196630 | 0,0731 |
| 42 | Thailand | Asiatic Region | 305015 | 104 | 0,0341 | 71702400 | 0,0145 |
| 43 | Ukraine | Eastern Europe | 302055 | 31 | 0,0103 | 37732800 | 0,0082 |
| 44 | Romania | Eastern Europe | 299401 | 98 | 0,0327 | 19118500 | 0,0513 |
| 45 | Argentina | Latin America | 297251 | 36 | 0,0121 | 45538400 | 0,0079 |
| 46 | Hungary | Eastern Europe | 268747 | 22 | 0,0082 | 9686460 | 0,0227 |
| 47 | Chile | Latin America | 244520 | 23 | 0,0094 | 19658800 | 0,0117 |
| 48 | Colombia | Latin America | 187928 | 73 | 0,0388 | 52321200 | 0,0140 |
| 49 | Nigeria | Africa | 175445 | 52 | 0,0296 | 227883000 | 0,0023 |
| 50 | Slovakia | Eastern Europe | 162757 | 15 | 0,0092 | 5518060 | 0,0272 |
| 51 | Croatia | Eastern Europe | 154324 | 18 | 0,0117 | 3896020 | 0,0462 |
| 52 | Serbia | Eastern Europe | 142159 | 104 | 0,0732 | 6773200 | 0,1535 |
| 53 | Viet Nam | Asiatic Region | 139043 | 87 | 0,0626 | 100352000 | 0,0087 |
| 54 | Slovenia | Eastern Europe | 136232 | 8 | 0,0059 | 2118400 | 0,0378 |
| 55 | Tunisia | Africa | 134434 | 77 | 0,0573 | 12200400 | 0,0631 |
| 56 | Iraq | Middle East | 126881 | 149 | 0,1174 | 45074000 | 0,0331 |
| 57 | United Arab Emirates | Middle East | 124488 | 70 | 0,0562 | 10642100 | 0,0658 |
| 58 | Morocco | Africa | 122005 | 34 | 0,0279 | 37712500 | 0,0090 |
| 59 | Bulgaria | Eastern Europe | 117142 | 10 | 0,0085 | 6795800 | 0,0147 |
| 60 | Algeria | Africa | 116123 | 45 | 0,0388 | 46164200 | 0,0097 |
| 61 | Bangladesh | Asiatic Region | 108707 | 82 | 0,0754 | 171467000 | 0,0048 |
| 62 | Jordan | Middle East | 82514 | 59 | 0,0715 | 11439200 | 0,0516 |
| 63 | Lithuania | Eastern Europe | 79503 | 8 | 0,0101 | 2854100 | 0,0280 |
| 64 | Philippines | Asiatic Region | 68595 | 49 | 0,0714 | 114891000 | 0,0043 |
| 65 | Ethiopia | Africa | 66363 | 231 | 0,3481 | 128692000 | 0,0179 |
| 66 | Peru | Latin America | 64597 | 33 | 0,0511 | 33845600 | 0,0098 |
| 67 | Estonia | Eastern Europe | 62678 | 5 | 0,0080 | 1367200 | 0,0366 |
| 68 | Kenya | Africa | 61619 | 13 | 0,0211 | 55339000 | 0,0023 |
| 69 | Lebanon | Middle East | 57050 | 24 | 0,0421 | 5773490 | 0,0416 |
| 70 | Qatar | Middle East | 55757 | 20 | 0,0359 | 2979080 | 0,0671 |
| 71 | Kazakhstan | Asiatic Region | 55445 | 123 | 0,2218 | 20330100 | 0,0605 |
| 72 | Belarus | Eastern Europe | 53486 | 14 | 0,0262 | 9115680 | 0,0154 |
| 73 | Cuba | Latin America | 52870 | 3 | 0,0057 | 11019900 | 0,0027 |
| 74 | Ecuador | Latin America | 51197 | 10 | 0,0195 | 17980100 | 0,0056 |
| 75 | Cyprus | Western Europe | 49781 | 9 | 0,0181 | 1344980 | 0,0669 |
| 76 | Venezuela | Latin America | 48729 | 6 | 0,0123 | 28300900 | 0,0021 |
| 77 | Ghana | Africa | 47904 | 32 | 0,0668 | 33787900 | 0,0095 |
| 78 | Latvia | Eastern Europe | 40648 | 5 | 0,0123 | 1882400 | 0,0266 |
| 79 | Kuwait | Middle East | 39905 | 23 | 0,0576 | 4838780 | 0,0475 |
| 80 | Sri Lanka | Asiatic Region | 39260 | 11 | 0,0280 | 22971600 | 0,0048 |
| 81 | Oman | Middle East | 37402 | 38 | 0,1016 | 5049270 | 0,0753 |
| 82 | Macao | Asiatic Region | 35359 | 6 | 0,0170 | 713912 | 0,0840 |
| 83 | Luxembourg | Western Europe | 34808 | 5 | 0,0144 | 665098 | 0,0752 |
| 84 | Iceland | Western Europe | 32773 | 5 | 0,0153 | 387558 | 0,1290 |
| 85 | Uganda | Africa | 32292 | 4 | 0,0124 | 48656600 | 0,0008 |
| 86 | Nepal | Asiatic Region | 31245 | 31 | 0,0992 | 29694600 | 0,0104 |
| 87 | Tanzania | Africa | 31237 | 4 | 0,0128 | 66617600 | 0,0006 |
| 88 | Georgia | Eastern Europe | 31114 | 8 | 0,0257 | 3807490 | 0,0210 |
| 89 | Uruguay | Latin America | 31011 | 5 | 0,0161 | 3388080 | 0,0148 |
| 90 | Uzbekistan | Asiatic Region | 30182 | 30 | 0,0994 | 35652300 | 0,0084 |
| 91 | Cameroon | Africa | 29343 | 10 | 0,0341 | 28372700 | 0,0035 |
| 92 | Armenia | Eastern Europe | 24346 | 11 | 0,0452 | 2943390 | 0,0374 |
| 93 | Azerbaijan | Eastern Europe | 24299 | 21 | 0,0864 | 10318200 | 0,0204 |
| 94 | Puerto Rico | Latin America | 22803 | 0 | 0,0000 | 3242020 | 0,0000 |
| 95 | Bosnia and Herzegovina | Eastern Europe | 22802 | 8 | 0,0351 | 3185070 | 0,0251 |
| 96 | Costa Rica | Latin America | 21425 | 1 | 0,0047 | 5105520 | 0,0020 |
| 97 | Zimbabwe | Africa | 18243 | 1 | 0,0055 | 16340800 | 0,0006 |
| 98 | North Macedonia | Eastern Europe | 18164 | 2 | 0,0110 | 1831800 | 0,0109 |
| 99 | Sudan | Africa | 17039 | 22 | 0,1291 | 50042800 | 0,0044 |
| 100 | Senegal | Africa | 15981 | 6 | 0,0375 | 18077600 | 0,0033 |

**Table 2. Number and proportion of retractions by country, and retractions per 10,000 inhabitants by country in the field of medicine (1996–2023). Countries sorted by total publication count.**

| Rank | Country | Region | Number of publications | Number of retractions | Retraction rate (%) | Population | Retractions per 10,000 inhabitants |
| --- | --- | --- | --- | --- | --- | --- | --- |
| 1 | United States | Northern America | 5464818 | 1216 | 0,0223 | 343477000 | 0,0354 |
| 2 | China | Asiatic Region | 2005477 | 3997 | 0,1993 | 1422580000 | 0,0281 |
| 3 | United Kingdom | Western Europe | 1548634 | 216 | 0,0139 | 68683000 | 0,0314 |
| 4 | Germany | Western Europe | 1252747 | 314 | 0,0251 | 84548200 | 0,0371 |
| 5 | Japan | Asiatic Region | 1143074 | 675 | 0,0591 | 124371000 | 0,0543 |
| 6 | Italy | Western Europe | 898294 | 251 | 0,0279 | 59499500 | 0,0422 |
| 7 | France | Western Europe | 845046 | 121 | 0,0143 | 66438800 | 0,0182 |
| 8 | Canada | Northern America | 795746 | 111 | 0,0139 | 39299100 | 0,0282 |
| 9 | Australia | Pacific Region | 649159 | 117 | 0,0180 | 26451100 | 0,0442 |
| 10 | Spain | Western Europe | 645565 | 122 | 0,0189 | 47911600 | 0,0255 |
| 11 | India | Asiatic Region | 631621 | 769 | 0,1218 | 1438070000 | 0,0053 |
| 12 | Netherlands | Western Europe | 526685 | 39 | 0,0074 | 18092500 | 0,0216 |
| 13 | Brazil | Latin America | 436692 | 69 | 0,0158 | 211141000 | 0,0033 |
| 14 | South Korea | Asiatic Region | 424886 | 274 | 0,0645 | 51748700 | 0,0529 |
| 15 | Turkey | Middle East | 369420 | 124 | 0,0336 | 87270500 | 0,0142 |
| 16 | Switzerland | Western Europe | 360967 | 37 | 0,0103 | 8870560 | 0,0417 |
| 17 | Sweden | Western Europe | 321392 | 46 | 0,0143 | 10551500 | 0,0436 |
| 18 | Belgium | Western Europe | 260824 | 26 | 0,0100 | 11712900 | 0,0222 |
| 19 | Poland | Eastern Europe | 258215 | 37 | 0,0143 | 38762800 | 0,0095 |
| 20 | Iran | Middle East | 243740 | 285 | 0,1169 | 90608700 | 0,0315 |
| 21 | Russian Federation | Eastern Europe | 229876 | 280 | 0,1218 | 145440000 | 0,0193 |
| 22 | Taiwan | Asiatic Region | 225163 | 76 | 0,0338 | 23317100 | 0,0326 |
| 23 | Denmark | Western Europe | 217557 | 23 | 0,0106 | 5948140 | 0,0387 |
| 24 | Austria | Western Europe | 180626 | 22 | 0,0122 | 9130430 | 0,0241 |
| 25 | Israel | Middle East | 166190 | 22 | 0,0132 | 9256310 | 0,0238 |
| 26 | Greece | Western Europe | 150027 | 51 | 0,0340 | 10242900 | 0,0498 |
| 27 | Norway | Western Europe | 146864 | 35 | 0,0238 | 5519170 | 0,0634 |
| 28 | Finland | Western Europe | 130776 | 9 | 0,0069 | 5601180 | 0,0161 |
| 29 | Mexico | Latin America | 125644 | 16 | 0,0127 | 129740000 | 0,0012 |
| 30 | Portugal | Western Europe | 119170 | 11 | 0,0092 | 10430700 | 0,0105 |
| 31 | Czech Republic | Eastern Europe | 118184 | 15 | 0,0127 | 10809700 | 0,0139 |
| 32 | South Africa | Africa | 114555 | 13 | 0,0113 | 63212400 | 0,0021 |
| 33 | Egypt | Africa/Middle East | 109553 | 233 | 0,2127 | 114536000 | 0,0203 |
| 34 | Hong Kong | Asiatic Region | 104678 | 16 | 0,0153 | 7442730 | 0,0215 |
| 35 | Saudi Arabia | Middle East | 104563 | 262 | 0,2506 | 33264300 | 0,0788 |
| 36 | New Zealand | Pacific Region | 103613 | 10 | 0,0097 | 5172840 | 0,0193 |
| 37 | Ireland | Western Europe | 101274 | 16 | 0,0158 | 5196630 | 0,0308 |
| 38 | Singapore | Asiatic Region | 95655 | 53 | 0,0554 | 5789090 | 0,0916 |
| 39 | Thailand | Asiatic Region | 93477 | 42 | 0,0449 | 71702400 | 0,0059 |
| 40 | Pakistan | Asiatic Region | 89973 | 146 | 0,1623 | 247504000 | 0,0059 |
| 41 | Argentina | Latin America | 84778 | 12 | 0,0142 | 45538400 | 0,0026 |
| 42 | Malaysia | Asiatic Region | 80785 | 58 | 0,0718 | 35126300 | 0,0165 |
| 43 | Hungary | Eastern Europe | 71852 | 12 | 0,0167 | 9686460 | 0,0124 |
| 44 | Chile | Latin America | 62102 | 2 | 0,0032 | 19658800 | 0,0010 |
| 45 | Romania | Eastern Europe | 60309 | 52 | 0,0862 | 19118500 | 0,0272 |
| 46 | Nigeria | Africa | 55970 | 12 | 0,0214 | 227883000 | 0,0005 |
| 47 | Colombia | Latin America | 54280 | 18 | 0,0332 | 52321200 | 0,0034 |
| 48 | Croatia | Eastern Europe | 48896 | 12 | 0,0245 | 3896020 | 0,0308 |
| 49 | Indonesia | Asiatic Region | 48493 | 17 | 0,0351 | 281190000 | 0,0006 |
| 50 | Serbia | Eastern Europe | 38990 | 25 | 0,0641 | 6773200 | 0,0369 |
| 51 | Slovakia | Eastern Europe | 34686 | 2 | 0,0058 | 5518060 | 0,0036 |
| 52 | Tunisia | Africa | 34622 | 19 | 0,0549 | 12200400 | 0,0156 |
| 53 | Ukraine | Eastern Europe | 30640 | 9 | 0,0294 | 37732800 | 0,0024 |
| 54 | Slovenia | Eastern Europe | 29856 | 3 | 0,0100 | 2118400 | 0,0142 |
| 55 | Bangladesh | Asiatic Region | 26780 | 38 | 0,1419 | 171467000 | 0,0022 |
| 56 | Morocco | Africa | 26767 | 17 | 0,0635 | 37712500 | 0,0045 |
| 57 | Ethiopia | Africa | 26119 | 52 | 0,1991 | 128692000 | 0,0040 |
| 58 | Bulgaria | Eastern Europe | 25537 | 5 | 0,0196 | 6795800 | 0,0074 |
| 59 | Kenya | Africa | 25306 | 6 | 0,0237 | 55339000 | 0,0011 |
| 60 | Cuba | Latin America | 25230 | 0 | 0,0000 | 11019900 | 0,0000 |
| 61 | United Arab Emirates | Middle East | 24887 | 25 | 0,1005 | 10642100 | 0,0235 |
| 62 | Lebanon | Middle East | 23460 | 14 | 0,0597 | 5773490 | 0,0242 |
| 63 | Viet Nam | Asiatic Region | 21693 | 18 | 0,0830 | 100352000 | 0,0018 |
| 64 | Peru | Latin America | 21543 | 5 | 0,0232 | 33845600 | 0,0015 |
| 65 | Iraq | Middle East | 21255 | 50 | 0,2352 | 45074000 | 0,0111 |
| 66 | Jordan | Middle East | 19283 | 30 | 0,1556 | 11439200 | 0,0262 |
| 67 | Uganda | Africa | 17683 | 2 | 0,0113 | 48656600 | 0,0004 |
| 68 | Qatar | Middle East | 17194 | 10 | 0,0582 | 2979080 | 0,0336 |
| 69 | Nepal | Asiatic Region | 17148 | 21 | 0,1225 | 29694600 | 0,0071 |
| 70 | Ghana | Africa | 16423 | 12 | 0,0731 | 33787900 | 0,0036 |
| 71 | Philippines | Asiatic Region | 15822 | 9 | 0,0569 | 114891000 | 0,0008 |
| 72 | Lithuania | Eastern Europe | 14957 | 2 | 0,0134 | 2854100 | 0,0070 |
| 73 | Tanzania | Africa | 14610 | 3 | 0,0205 | 66617600 | 0,0005 |
| 74 | Venezuela | Latin America | 14225 | 3 | 0,0211 | 28300900 | 0,0011 |
| 75 | Kuwait | Middle East | 11552 | 9 | 0,0779 | 4838780 | 0,0186 |
| 76 | Sri Lanka | Asiatic Region | 11479 | 7 | 0,0610 | 22971600 | 0,0030 |
| 77 | Ecuador | Latin America | 11288 | 4 | 0,0354 | 17980100 | 0,0022 |
| 78 | Cyprus | Western Europe | 11210 | 3 | 0,0268 | 1344980 | 0,0223 |
| 79 | Estonia | Eastern Europe | 10894 | 0 | 0,0000 | 1367200 | 0,0000 |
| 80 | Iceland | Western Europe | 10618 | 2 | 0,0188 | 387558 | 0,0516 |
| 81 | Cameroon | Africa | 10306 | 6 | 0,0582 | 28372700 | 0,0021 |
| 82 | Oman | Middle East | 9834 | 10 | 0,1017 | 5049270 | 0,0198 |
| 83 | Algeria | Africa | 9095 | 4 | 0,0440 | 46164200 | 0,0009 |
| 84 | Uruguay | Latin America | 9025 | 3 | 0,0332 | 3388080 | 0,0089 |
| 85 | Bosnia and Herzegovina | Eastern Europe | 8279 | 1 | 0,0121 | 3185070 | 0,0031 |
| 86 | Malawi | Africa | 8116 | 1 | 0,0123 | 21104500 | 0,0005 |
| 87 | Puerto Rico | Latin America | 7950 | 0 | 0,0000 | 3242020 | 0,0000 |
| 88 | Luxembourg | Western Europe | 7947 | 4 | 0,0503 | 665098 | 0,0601 |
| 89 | Georgia | Eastern Europe | 7620 | 1 | 0,0131 | 3807490 | 0,0026 |
| 90 | Belarus | Eastern Europe | 7448 | 5 | 0,0671 | 9115680 | 0,0055 |
| 91 | Senegal | Africa | 7002 | 4 | 0,0571 | 18077600 | 0,0022 |
| 92 | Sudan | Africa | 6641 | 16 | 0,2409 | 50042800 | 0,0032 |
| 93 | Macao | Asiatic Region | 6587 | 1 | 0,0152 | 713912 | 0,0140 |
| 94 | Zimbabwe | Africa | 6522 | 1 | 0,0153 | 16340800 | 0,0006 |
| 95 | Zambia | Africa | 5984 | 0 | 0,0000 | 20724000 | 0,0000 |
| 96 | Kazakhstan | Asiatic Region | 5880 | 12 | 0,2041 | 20330100 | 0,0059 |
| 97 | Latvia | Eastern Europe | 5619 | 0 | 0,0000 | 1882400 | 0,0000 |
| 98 | Burkina Faso | Africa | 5584 | 0 | 0,0000 | 23025800 | 0,0000 |
| 99 | Costa Rica | Latin America | 5278 | 0 | 0,0000 | 5105520 | 0,0000 |
| 100 | Bahrain | Middle East | 5010 | 24 | 0,4790 | 1569670 | 0,1529 |

**Criteria for retraction used in the Retraction Watch Database**

+Author Unresponsive OR +Breach of Policy by Author OR +Concerns/Issues About Authorship OR +Conflict of Interest OR +Duplication of Article OR +Duplication of Data OR +Duplication of Image OR +Duplication of Text OR +Ethical Violations by Author OR +Fake Peer Review OR +Falsification/Fabrication of Data OR +Falsification/Fabrication of Image OR +Falsification/Fabrication of Results OR +False/Forged Authorship OR +Informed/Patient Consent - None/Withdrawn OR +Lack of Approval from Author OR +Lack of IRB/IACUC Approval OR +Manipulation of Images OR +Manipulation of Results OR +Miscommunication by Author OR +Misconduct by Author OR +Nonpayment of Fees/Refusal to Pay OR +Original Data not Provided OR +Paper Mill OR +Plagiarism of Article OR +Plagiarism of Data OR +Plagiarism of Image OR +Plagiarism of Text OR +Randomly Generated Content OR +Sabotage of Materials OR +Salami Slicing
